# Medical Decision‐Making Capacity in Early‐Stage Alzheimer Patients: Comparison With Healthy Controls and Benefit of Assistive Information

**DOI:** 10.1111/psyg.70227

**Published:** 2026-10-14

**Authors:** Dilara Aylin Felice Vossberg, Vasilija Rolfes, Heiner Fangerau, Martin Haupt

**Affiliations:** ^1^ Department of the History, Philosophy and Ethics of Medicine, Medical Faculty Heinrich‐Heine‐Universität Düsseldorf, Universitätsstraße 1 Düsseldorf Germany; ^2^ Teaching Practice of the Clinic and Polyclinic for Psychiatry and Psychotherapy, Medical Faculty Heinrich‐Heine‐Universität Düsseldorf; Hohenzollernstraße 5 Düsseldorf Germany

**Keywords:** Alzheimer disease, consent, decision‐making capacity, patients, visual aids

## Abstract

**Background:**

Early‐stage Alzheimer Disease (AD) may lead to restricted decision‐making capacity and without standardized instruments, there is great variability in determining patients' ability to give consent. This study aimed to assess decision‐making capacity of AD patients in comparison to a healthy control group and investigated the usefulness of visual aids.

**Methods:**

A group of 30 ad patients and 30 healthy controls were compared using the MacArthur Competence Assessment Tool for Treatment (MacCAT‐T). Additionally, we implemented a modified version of the MacCAT‐T with visual aids.

**Results:**

Results revealed a significant difference in the MacCAT‐T domain of Understanding between the AD and control group after adjustment for age, sex, and education. No significant differences were found for Appreciation, Reasoning, or Expression of Choice. Adding visual aids led to no significant improvements.

**Conclusions:**

Standardized assessments can be valuable tools for determining the decision‐making capacity of patients with early‐stage AD. The efficacy of visual aids should be further investigated.

## 1 Introduction

Alzheimer Disease (AD) is the most common form of dementia worldwide, attracting increasing medical and research interest over recent decades. The origin of the disease is not yet fully understood and a cure has not been found [1]. Even in its initial symptomatic stages, confusion, difficulties with speaking and changes in personality and behaviour may occur [2]. There are no standardized procedures to govern the elicitation of decision‐making capacity about medical interventions; evaluating the extent to which an Alzheimer patient is capable of giving consent is up to the treating physician, which can lead to large interindividual differences in what counts as decision‐making capacity [3]. This capacity must be assessed to ensure the patient's greatest possible autonomy during diagnostics and medical treatment. At the same time, patients' medical decision‐making capacity should not be overestimated. If patients no longer have the capacity to provide informed consent because of cognitive impairment, they may make decisions that could cause them harm and that may not reflect their values and preferences [4, 5]. As this may conflict with the ethical principle of non‐maleficence, a thorough assessment using a standardized procedure is particularly valuable in medical settings [6].

When patients are no longer able to make decisions, ethical guidelines and laws must be followed to ensure that their rights and dignity are preserved [7], which is especially important for patients in the early stages of AD. They often experience feelings of helplessness and uncertainty and may also suffer financial losses due to impaired financial capacity [8, 9]. These factors may in turn be associated with the development of depression or other psychiatric disorders [10]. To make a comprehensive assessment of patients' ability to provide informed consent, it is important to consider not only cognitive functioning, but also mental health, functional abilities, individual values and risks associated with the specific decision. Furthermore, as outlined in the guidelines of the American Bar Association Commission on Law and Aging and the American Psychological Association, potential means of enhancing patients' decision‐making capacity should be considered before reaching a final judgement [11, 12]. According to the German S2k guideline on informed consent of people with dementia in medical interventions, a thorough and individualized assessment is needed to ensure that patients with dementia receive appropriate support to maximize their ability to participate in medical decision‐making [13].

Using standardized instruments promotes equity and procedural fairness in clinical practice, as it helps ensure that all patients are evaluated comprehensively and according to consistent criteria, thereby reducing variability in the assessment process [14]. Various instruments have been used to assess decision‐making capacity. For example, Okonkwo et al. used the Capacity to Consent to Treatment Instrument, a conceptually based tool, to investigate medical decision‐making capacity in healthy controls, patients with mild cognitive impairment, and patients with mild AD and found impaired decision‐making capacity in both patient groups [15]. A systematic review by Sessums et al., which also included studies involving patients with AD, summarized findings across different assessment instruments and reported a substantially higher prevalence of impaired decision‐making capacity in clinical populations compared with healthy elderly control participants. However, the authors also noted heterogeneity across studies regarding the instruments used and the severity of cognitive impairment [16]. As a further example, financial capacity, another aspect of decision‐making capacity that is often diminished in patients with cognitive impairment, can be assessed using the Financial Capacity Instrument [8].

However, one of the best‐known methods for standardized assessment of capacity to consent is the MacArthur Competence Assessment Tool for Treatment (MacCAT‐T). The MacCAT‐T assesses four dimensions of what is perceived as capacity to consent: 1. Understanding (disclosed information), 2. Appreciation (of the significance of the disclosed information for one's own situation), 3. Reasoning (about risks and benefits of one's choices and treatment options), and 4. Expression of Choice (regarding a proposed treatment) [17]. According to the original authors, scores on the individual MacCAT‐T domains should not serve as diagnostic criteria for determining decision‐making capacity and no validated cut‐off scores have been established. Higher scores within a domain nevertheless indicate greater decision‐making capacity [17, 18, 19]. The MacCAT‐T enables a thorough assessment of an Alzheimer patient's ability to provide informed consent to medical treatment or research. It is a well‐researched, well‐evaluated instrument that can be applied in multiple research areas, is used in patients with different mental and neurodegenerative diseases, and has been adapted for use across different countries and languages [20, 21]. For example, it has been applied in an international study with schizophrenic patients across multiple European countries to test whether they showed more capacity to consent in a forensic placement or during an inpatient hospital stay [22].

In addition, the semi‐structured interview can be designed differently in terms of content depending on the patient group and it can also be enhanced with aids intended to make it easier for the interviewees to understand the given information. Support measures can include, but are not limited to, the use of visual aids, written information, simplified language, extended time for decision‐making based on individual needs, environments free of distraction or involvement of trusted persons [13]. One model recently published by Scholten, Gather and Vollmann acknowledges the challenges associated with decision‐making capacity assessment and proposes a combined supported decision‐making approach that incorporates appropriate support measures into the assessment process, thereby providing a theoretical rationale for the use of aids during capacity assessments. The model focuses on patient autonomy and specifically emphasizes the use of support measures prior to the assessment of decision‐making capacity. At the same time, the authors caution that evidence regarding the effectiveness of such support measures remains limited [23]. Mittal et al. [24] provided participants with AD and mild cognitive impairment with either a slideshow presentation or an enhanced version of the consent form before testing them with a modified MacArthur Competence Assessment Tool for Clinical Research (MacCAT‐CR). Both groups showed the same level of understanding with verbal re‐explanation being associated with higher scores in Understanding. Palmer et al. [14] found the use of multimedia tools not to significantly impact the consent process for mild and moderate Alzheimer patients. Dunn and Jeste [25] postulated that understanding of informed consent itself could be strengthened by feedback with corrections, multiple learning trials, and simplified consent forms. Studies on the usefulness of visual aids for older adults have shown mixed results, which is why this work is an important addition to the current field of research. For example, Liu et al. reported that the use of illustrations led to confusion among elderly patients rather than improving their understanding [26]. On the other hand, Garcia‐Retamero and Cokely found visual aids to be effective tools for improving decision‐making [27]. As outlined by Poth et al., several other studies have reported positive effects associated with the use of visual aids [28]. These examples reflect how the present state of research is still heterogeneous in its attempts to enhance Alzheimer patients' capacity to give informed consent. However, the present study focused on the application of the MacCAT‐T in early‐stage AD to expand upon existing research in this field.

Although the four MacCAT‐T domains represent distinct components of decision‐making capacity, they are not necessarily independent of one another. Understanding the given information may facilitate reflection on the disclosed content, its application to one's own situation and the ability to provide reasons for a chosen treatment option. The communication of a treatment choice may also be influenced by these processes [29, 30, 31]. To evaluate the relevance of the domains, all four are therefore included in the present study. The first aim of this work is to compare, using the MacCAT‐T, the decision‐making capacity between early‐stage AD patients and a control group of comparable age with no signs of cognitive impairment. Accordingly, our hypothesis is that the consent capacity of patients with early‐stage AD differs significantly across the four MacCAT‐T domains from the control group when using the instrument. Our second aim is to examine whether enhancing the informed consent process with visual aids during the information disclosure affects performance across the MacCAT‐T domains in the AD group. We hypothesize that patients with early‐stage AD would benefit from these aids and that they would achieve higher MacCAT‐T scores across the domains than patients with early‐stage AD who did not receive the visual aids.

## 2 Methods

### 2.1 Participants

A total of 62 subjects participated in the study. Participants were selected to be between 50 and 100 years of age. All subjects in the AD group were recruited through a private psychiatric practice (MH) in Düsseldorf, Germany. Patients attending the practice were consecutively invited to participate in the study. Controls without AD were recruited among patients' relatives and through the personal networks of the research team. Diagnoses were made according to the diagnostic criteria for Alzheimer Dementia of the ICD‐10 [32]. All patients were interviewed and participated in a physical status examination, magnetic resonance imaging (MRI), and cognitive testing. Included in the study were all patients with early‐stage AD showing symptoms of mild cognitive impairment and mild dementia. None of the AD patients suffered from depression or anxiety disorder, as psychiatric disorders were an exclusion criterion. Furthermore, there were no clinically relevant behavioural or psychological symptoms within this group; therefore, psychopharmacological medication did not have to be prescribed. Relatives of the participating patients were asked whether they would also be willing to take part in the study as control subjects. In addition, some control subjects were externally recruited. Control group participants showed no signs or symptoms of any form of dementia. AD patients with moderate or severe AD were excluded; patients suffering from other forms of dementia or other neurological disorders were also excluded. Due to their young age and the onset of dementia, two subjects from the control group had to be excluded from the study, leaving a total sample size of 60 participants.

### 2.2 Procedures

All cognitive tests and MacCAT‐T interviews were conducted by two trained members of the research team (DV, MH). To ensure appropriate interviewing, briefings to the two members were given with the aid of exemplary video recordings. All participants were interviewed with an adapted MacCAT‐T version, which was an alteration of the original MacCAT‐T specifically adjusted to the group of AD patients and based on the German translation by Jochen Vollmann [33, 34]. While there is now a more recent standardized and validated German translation available, the version used was the most recent version at the time of data collection. The updated translation provides comprehensive administration and scoring materials as well as guidance for research and clinical use [35]. In the present study, one half of the AD group received a modified version of the MacCAT‐T, which contained three additional supporting items in the following order: a cranial MRI with pictures focusing on global cerebral and on hippocampal involution signs (Figure S1), a diagram showing the effect of the antidementia medication offered (Figure S2), and the antidementia medication itself in its original packaging. The MRI image visually illustrated the current effects of the disease, the diagram showed disease progression with its long‐term consequences, and the medication box provided a real‐life reference that could be touched. Visual aids were intentionally chosen to represent common items or graphs that patients could relate to and that were commonly used in clinical counselling contexts. All aids were piloted for comprehension in the private practice. The AD patients who were shown the items prior to study data collection all rated the aids as helpful and appeared to demonstrate an adequate understanding of the information during pretesting. Each item was presented for an average of two to 3 min, but the presentation time could extend up to 10 min, depending on when patients demonstrated an understanding of the item. AD patients were randomly assigned to the groups with and without the modified version. The control group and the AD group were tested within the same time period. All groups received identical information about AD and an option for a hypothetical treatment during the course of the semi‐structured MacCAT‐T interview. The AD group with the modified tool was additionally presented with the three visual aids. Explanations were given if necessary; additional time was provided for questions to be asked and answered. The effectiveness of the visual aids was then evaluated by comparing the two AD groups.

### 2.3 Instruments

Cognitive testing consisted of five different measures and the clinician's judgement with respect to the clinical dementia rating (CDR) score was given. The DemTect test systematically examines a range of cognitive functions [36]: recall, including delayed recall, of a word list, the backward digit span, and the semantic word fluency tasks. First, participants had to remember 10 words and reproduce them immediately in two trials. Then they were asked to name up to 30 different animals within 1 min. After reciting a series of up to 6 numbers backwards, they were asked to recall the initial 10 words [37]. The remaining two cognitive tests consisted of the Constructional Praxis tasks, as measured by the Alzheimer disease assessment scale (ADAS), presenting four different geometric figures and the trail making test (TMT), parts A and B [38, 39]. For the word list, achievable scores ranged from 0 to 10, for the working memory task from 0 to 6 and for the fluency task from 0 to 30. Visuo‐construction tasks in the ADAS resulted in scores ranging from 0 to 11 and the worst score for the TMT tests was a processing time of 240 s. The clinician's judgement on the overall severity of cognitive symptoms was based on the CDR Instrument. Its score is composed of six categories: Memory, Orientation, Judgement and Problem Solving, Community Affairs, Home and Hobbies and Personal Care. The CDR score can range from 0 to 3. We only included scores from 0 to 1, with 0 indicating no impairment, 0.5 representing questionable or very mild dementia and a value of 1 reflecting more pronounced cognitive decline and symptoms of mild AD. As all patients included in the study were diagnosed with early‐stage AD, the CDR score was used to indicate the severity of impairment rather than to establish the diagnosis [40, 41].

Decision‐making capacity was evaluated using the adapted MacCAT‐T, following Grisso's and Appelbaum's work. Accordingly, the four decision abilities Understanding, Appreciation, Reasoning, and Expression of Choice were included. Full points for Understanding were given if the person could repeat information relating to disease, treatment, as well as risks and benefits. For the next two sections, Appreciation of the disease and treatment was asked about, and the Reasoning conversation was subdivided into questions to assess consequential and comparative thinking, as well as the generation of consequences. Finally, by Expressing a Choice, individuals could indicate which treatment option they preferred. Subsection scores of the MacCAT‐T range from 0 to 6 for Understanding, 0 to 4 for Appreciation, 0 to 8 for Reasoning, and 0 to 2 for Expressing a Choice [17].

### 2.4 Statistical Analysis

Statistical analysis was done using the IBM SPSS Statistics software (version 29.0.0.0) with the level of significance at *p* ≤ 0.05 for two‐tailed tests to account for effects in both directions. To describe the demographic data, mean and standard deviation (SD) were used for parametric quantities and frequencies and percentages for nonparametric data. Correlations were quantified with the Spearman coefficient, interpreted by Cohen's criteria (*r* < 0.30 is a weak correlation, *r* within 0.30–0.49 suggests a moderately strong correlation, *r* > 0.50 indicates a strong correlation) [42]. For all inter‐group comparisons of the four MacCAT‐T domains, general linear models were used, adjusting for age, sex and education. The homogeneity of regression slopes and homogeneity of error variances were assessed using interactions between group and age and Levene's test. Partial *η* ^*2*^ was reported as a measure of effect size. Post hoc power analyses were conducted using G*Power 3.1 [43].

## 3 Results

Sociodemographic data for the AD and control group are depicted in Table 1. The total sample consisted of 60 participants with 30 belonging to the early‐stage AD group and 30 to the control group. The AD and control group exhibited substantial and statistically significant differences in age, education, and sex, and similarity in the lifestyle measures. The CDR scores reflected the expected differences between the groups. The Alzheimer patients had a mean age of 76 years (SD = 9.27), the control group of 69 years (SD = 9.36). Sixty percent of the control group were female, while 73% of the AD group were male. More people in the AD group were university graduates and therefore achieved a higher level of education (53%) than in the control group (13%). The number of people who had been employed during their lifetime was the same in both groups (96%) as well as the number of people being married or having a partner (70%). One more participant of the control group (20%) lived alone compared to the AD group (17%). In the early‐stage AD group, CDR scores of 0.5 or 1 were given, with an average score of 0.73 (SD = 0.25) for all 30 patients. All participants in the control group had a CDR score of 0 with no cognitive impairments.

**TABLE 1:** Sociodemographic data of AD patients and control group.

Variable	AD group (*n* = 30)	Control group (*n* = 30)	*p*
Age	76 (9.3)	69 (9.4)	0.004*
Sex (% women)	8 (26.7)	18 (60)	0.009*
University graduate (% achieved)	16 (53.3)	4 (13.3)	< 0.001*
Employed (% working)	29 (96.7)	29 (96.7)	1.000
Has partner (% married/partner)	21 (70)	21 (70)	1.000
Living situation (% alone)	5 (16.7)	6 (20)	0.744
CDR score	0.73 (0.3)	0*	< 0.001*

* Mean (SD) difference is significant at the 0.05 level (2‐tailed).

Correlations between cognitive tests and the four MacCAT‐T domains in the AD group are shown in Table 2. Understanding correlated with several cognitive tests; in other MacCAT‐T domains only one correlation was found for each of Appreciation and Reasoning. Strong correlations with Understanding were found for the semantic word fluency task (*r* = 0.61, *p* < 0.001) and the CDR score (*r* = −0.66, *p* < 0.001). Understanding also correlated moderately with delayed word list recall (*r* = 0.42, *p* = 0.021). In the other MacCAT‐T domains, moderate correlations were found for Appreciation with TMT A (*r* = −0.44, *p* = 0.015) and Reasoning with ADAS visuoconstruction (*r* = 0.42, *p* = 0.022).

**TABLE 2:** Correlations of cognitive tests and CDR score with MacCAT‐T domains in the AD group.

Cognitive tests	Understanding		Appreciation		Reasoning		Expression of choice	
	*r*	*p*	*r*	*p*	*r*	*p*	*r*	*p*
Word list	0.36	0.054	0.13	0.491	0.26	0.169	0.10	0.625
Word list delayed	0.42	0.021*	0.20	0.298	0.07	0.735	0.33	0.074
Backward digit span	0.35	0.058	−0.23	0.219	0.11	0.578	0.25	0.191
Semantic word fluency	0.61	< 0.001*	0.15	0.431	0.34	0.064	0.15	0.421
Visuoconstruction ADAS	0.14	0.448	0.05	0.798	0.42	0.022*	0.30	0.108
TMT A	−0.26	0.162	−0.44	0.015*	−0.15	0.435	0.16	0.413
TMT B	−0.12	0.543	−0.18	0.354	−0.31	0.098	−0.19	0.319
CDR score	−0.66	< 0.001*	−0.20	0.293	−0.29	0.121	−0.33	0.071

*Mean (SD) difference is significant at the 0.05 level (2‐tailed).

To test our first hypothesis, results are presented in Table 3. We assumed that our version of the MacCAT‐T, which was adapted to Alzheimer patients with early‐stage AD, would be able to differentiate significantly across the domains between the AD and control group. As predicted, we found a significant difference between the two groups in one of the MacCAT‐T domains. After adjustment for age, sex and education, examination of the individual MacCAT‐T domains showed that there was a significant difference between patients and the control group within the domain Understanding (*F*[1,55] = 4.90, *p* = 0.031, partial *η* ^*2*^ = 0.082). The control group showed slightly higher mean values (*M* = 5.50, SD = 0.54) in comparison to the AD group (*M* = 5.08, SD = 0.64). No significant differences were found for the domains Appreciation (*F*[1,55] = 0.99, *p* = 0.324, partial *η* ^*2*^ = 0.018), Reasoning (*F*[1,55] = 2.08, *p* = 0.155, partial *η* ^*2*^ = 0.036) and Expression of Choice (*F*[1,55] = 2.09, *p* = 0.154, partial *η* ^*2*^ = 0.037).

**TABLE 3:** Adjusted comparison of MacCAT‐T results.

	AD group (*n* = 30)	Control group (*n* = 30)	*F‐*value^a^	df^a^	*p* ^a^	Partial *η* ^*2*^ ^a^	Power
Understanding	5.08 (0.64)	5.50 (0.54)	4.90	1, 55	0.031*	0.082	0.624
Appreciation	3.97 (0.18)	4.00 (0.00)	0.99	1, 55	0.324	0.018	0.177
Reasoning	7.27 (1.11)	7.53 (1.01)	2.08	1, 55	0.155	0.036	0.312
Expression of choice	1.87 (0.43)	1.97 (0.18)	2.09	1, 55	0.154	0.037	0.320

^a^ Group effects were adjusted for age, sex and education.

* Mean (SD) difference is significant at the 0.05 level (2‐tailed).

Findings concerning our second hypothesis are shown in Table 4. We hypothesized that the decision‐making capacity of patients with early‐stage AD could be improved by using an enhanced version of the MacCAT‐T tool. However, after adjustment for age, sex and education, the individual MacCAT‐T domains Understanding (*F*[1,25] = 2.35, *p* = 0.137, partial *η* ^*2*^ = 0.086), Appreciation (*F*[1,25] = 0.60, *p* = 0.446, partial *η* ^*2*^ = 0.023), Reasoning (*F*[1,25] = 0.48, *p* = 0.495, partial *η* ^*2*^ = 0.019), and Expression of Choice (*F*[1,25] = 0.16, *p* = 0.689, partial *η* ^*2*^ = 0.007) did not show statistically significant differences with the use of the modified version of the MacCAT‐T.

**TABLE 4:** Adjusted comparison of standard and modified MacCAT‐T tool.

	Standard MacCAT‐T (*n* = 16)	Modified MacCAT‐T (*n* = 14)	*F* value^a^	df^a^	*p* ^a^	Partial *η* ^*2*^ ^a^	Power
Understanding	5.27 (0.54)	4.85 (0.69)	2.35	1, 25	0.137	0.086	0.366
Appreciation	4.00 (0.00)	3.93 (0.27)	0.60	1, 25	0.446	0.023	0.127
Reasoning	7.50 (0.89)	7.00 (1.30)	0.48	1, 25	0.495	0.019	0.113
Expression of choice	1.94 (0.25)	1.79 (0.58)	0.16	1, 25	0.689	0.007	0.073

^a^ Group effects were adjusted for age, sex and education.

In summary, the results show that decision‐making capacity in patients with early‐stage AD differed significantly from that of the control group only in the Understanding domain. These findings remained unchanged after adjusting for age, sex, and education. The use of the three visual aids included in this study did not lead to significant improvements in the early‐stage AD group that received the modified version of the MacCAT‐T.

## 4 Discussion

In the present study, the MacCAT‐T was adapted to patients with AD and used to investigate the differences in the ability to consent between patients with early‐stage AD and an unimpaired (healthy) control group. The results regarding our first hypothesis, which stated that the AD and control groups would differ significantly in their consent capacity across the four domains, show that the instrument may reliably distinguish between the two groups through one of the domains. Patients with AD achieved significantly lower scores in the Understanding domain of the MacCAT‐T. This finding adds to the existing literature. Impairments in decision‐making capacity and the understanding of information have also been identified using various other instruments [44, 45]. In particular, executive functioning and processing speed were identified as relevant predictors of impaired decision‐making capacity in patients with mild AD by Okonkwo et al. The same study found appreciation and reasoning to be impaired when assessed using the Capacity to Consent to Treatment Instrument [45]. Using the MacCAT‐T, however, the present study found no significant differences between patients with early‐stage AD and healthy controls in the appreciation of the disease and treatment or in the ability to reason or to make a choice. Correlations between MacCAT‐T and cognitive tests supported our findings, showing that different tasks correlated strongly with the domain Understanding, while for all other domains of the MacCAT‐T correlations remained low to absent. Correlations were found for the Understanding domain with the word list delayed recall task and semantic word fluency, as measured by the cognitive examination. Each of these two cognitive functions requires the storage and recall of verbal information. It is well known that Alzheimer patients in the early stage of the disease experience severe difficulties in retaining information. Therefore, these results are consistent with the clinical experience that patients with early‐stage AD often show limitations in short‐term information intake, while general reflective and processing abilities are mainly preserved [46].

The interpretation of our results requires consideration of the sociodemographic composition of the AD group relative to the control group, as the AD group consisted predominantly of privately insured patients, which may indicate a higher socioeconomic status. This difference was also supported by the sociodemographic data: 53% of the patients were university graduates, while only 13% of the control group had received that level of education. It may be anticipated, therefore, that the AD group would score better in terms of their decision‐making ability and especially their understanding compared to our control group due to their higher socioeconomic status. This assumption was not confirmed by the data, as the analyses revealed no evidence consistent with the expected pattern. In addition, statistically significant group differences were still found with the control group achieving better results in cognitive tasks. Presupposing the association of higher socioeconomic status with decision‐making capacity, one may conclude from these findings that, even in patients with early‐stage AD, there may already be an impairment in the ability to consent. This highlights the need for more standardized and thorough procedures to identify decision‐making ability in the early stages of the disease. A longitudinal study could help to examine the effects of AD on decision‐making capacity over a longer period of time and complement the results of this work. A prerequisite for such studies is the use of equivalent assessment versions or counterbalancing to improve the validity of longitudinal assessments, as decision‐making capacity may vary depending on the assessment scenario [47].

The MacCAT‐T has been shown to be advantageous in clinical practice, and this work further demonstrated that it can differentiate well for decision‐making capacity between a group of patients with early‐stage AD and a healthy control group. An advantage of this study is the implementation of the MacCAT‐T in a clinical setting with a homogeneous sample in the very mild stages of AD compared to other studies that mixed patients with mild and moderate dementia severity [14, 29]. Our results underline the need for a thorough examination of decision‐making capacity during the early stages of AD as some patients already showed significantly impaired cognitive processing abilities in the MacCAT‐T, while others did not.

Because the MacCAT‐T has a strong cognitive focus, it should only be used in combination with an experienced physician's medical judgement. In this study, the CDR score may have been comparable to a physician's clinical assessment. As our results showed, there were some close correlations between the MacCAT‐T scores and the physician's clinical assessment. However, not all domains of the MacCAT‐T correlated significantly with the CDR score. Therefore, a standardized instrument may be used as a complementary method to assess capacity to consent for patients with early‐stage AD.

In accordance with our findings, previous studies have also shown that, especially for the Understanding domain, lower scoring is achieved by patients with mild cognitive deficits [48]. Therefore, our second hypothesis suggested that our modified version of the MacCAT‐T with additional assistive information would lead to better MacCAT‐T results in Understanding and, presumably, in other domains as well. To our knowledge, this has been the first study to investigate the benefit of visual aids in combination with the MacCAT‐T in a homogeneous group of early‐stage AD patients. In contrast to our expectation, we found that the modified MacCAT‐T showed no advantage over the conventional interview. This raises the question of whether enhancements or aids to the informed consent process provide any benefits in this case, especially considering that the patients were in the very mild stages of AD. It is possible that our material was too distracting or overwhelming as we used a medication box, an MRI image and a diagram presentation. Especially the latter two visual aids might have been difficult to understand—even with explanations provided—for patients with cognitive deficits. However, this does not mean that assistive materials are generally not helpful. Different aids were used in other studies in which supportive materials were found to be helpful [7]. One systematic review by Nishimura et al. found that especially extended discussions or enhanced consent forms were useful aids for improving the understanding of information [49]. The findings by Poth et al. further suggest the selection of supportive aids based on individual patients' preferences and needs [28]. While the authors underline the importance of simple aids adapted to the individual patient, Chen et al. found that, particularly in healthcare‐related contexts, complex and interactive aids were mostly used for older adults with cognitive impairment [50]. This further highlights a potential discrepancy between the frequency with which certain aids are used and their actual effectiveness. At the same time, simpler aids may be more suitable for facilitating understanding in elderly patients with early‐stage AD [51]. Furthermore, one needs to consider the severity of AD in the patient and what type of assistive devices are most appropriate [28]. Aids that are easier to understand, such as pictures or simple texts to read, may work better than more complex aids such as an MRI, even for patients with early‐stage AD. Mueller et al., for example, explored enhancement options for the MacCAT‐T for patients with mild to moderate AD in their study and pointed out that it is important to mostly give basic information to patients with AD and that enhancements should focus on reducing attention and memory demands [29].

A limitation of this study relates to the small sample size, due to data collection during the COVID‐19 pandemic period when all interactions with elderly people were greatly reduced. The limited statistical power may increase the risk of Type II error, and especially the non‐significant findings for the second hypothesis should not be interpreted as evidence of the absence of an effect. Participants were recruited from a private psychiatric practice specializing in the treatment of patients with mild dementia, which may limit the representativeness of the study sample. Although the adapted German version of the MacCAT‐T was based on the validated translation by Vollmann [33], the most recent German version available at the time, the findings may not be directly generalizable to other cultural settings [20]. In addition, two raters were involved in the evaluation of the MacCAT‐T, which may have led to differences in scoring. As each case was scored only once, inter‐rater reliability could not be assessed. To minimize potential differences in scoring, ambiguous cases were discussed between raters to reach agreement.

In the future, it would be important to investigate whether other modified versions of the MacCAT‐T may provide more clinically relevant results. On the other hand, however, it may be thought that our type of modified information was not suitable for assisting AD patients with very mild forms of dementia symptoms. Cognitive capabilities might, for example, vary in this group of patients and result in different MacCAT‐T scores [48]. Future studies should explore the relevance of simpler pictures, texts, or other kinds of enhancements for the informed consent process in relation to different stages of the disease.

Despite its limitations, this study brings new insights into the field of informed consent with respect to Alzheimer patients with mild dementia. Our adapted version of the MacCAT‐T has proven to be a useful tool for distinguishing between very mildly affected AD patients and healthy controls. Regular use of the MacCAT‐T can, therefore, help monitor the progression of AD and reveal a change in the ability to consent. This is particularly relevant in the early stages of AD, as it is usually only during or after these mild stages that a decrement of capacity to consent occurs [15].

## Funding

The present study was supported by a grant from the Alzheimer Society of Germany (Deutsche Alzheimer Gesellschaft). Grant/Award Number: 2020‐1061. The funding source had no role in the study design, data analysis, interpretation of the results or writing of the report.

## Ethics Statement

The study was approved by the Ethics Committee of the Heinrich‐Heine‐Universität Düsseldorf. Informed consent documents were signed by participants after having received both oral and written information about the study aims and methods.

## Consent

All human subjects provided informed consent prior to and during the course of the study.

## Conflicts of Interest

The authors declare no conflicts to interest.

## Supporting information


**Figure S1:** MRI scan of head—anonymized patient, provided by MH.
**Figure S2:** Chart produced by MH for patient information.

## Data Availability

The datasets generated and analysed during the current study are available from the corresponding author upon request.
