# Supplementary material for: Medical Decision‐Making Capacity in Early‐Stage Alzheimer Patients: Comparison With Healthy Controls and Benefit of Assistive Information
Source: Psychogeriatrics. 2026 Oct 14;26(6):e70227. doi: 10.1111/psyg.70227 (PMC13662617; doi:10.1111/psyg.70227)
Supplement: Supplementary file 1 [file PSYG-26-0-s001.docx]

**Supporting Information**

**FIGURE 1.** MRI scan of head – anonymized patient, provided by MH

**FIGURE 2.** Chart produced by MH for patient information

Format: Word (.doxc)

**FIGURE 1.** MRI scan of head – anonymized patient, provided by MH

Hippocampal atrophy	Generalized cerebral atrophy

**FIGURE 2.** Chart produced by MH for patient information
